# Public Mass Shootings: Counterfactual Trend Analysis of the Federal Assault Weapons Ban

**DOI:** 10.2196/62952

**Published:** 2024-09-20

**Authors:** Alex Lars Lundberg, James Alan Fox, Hassan Mohammad, Maryann Mason, Doreen Salina, David Victorson, Ruben Parra-Cardona, Lori Ann Post

**Affiliations:** 1 Buehler Center for Health Policy & Economics, Feinberg School of Medicine, Northwestern University, 9-933 Rubloff Building, 420 E Superior St, Chicago, IL, 60611, United States, 1 312-503-4559; 2 Criminology, Law and Public Policy, Northeastern University, New York, NY, United States; 3 Department of Psychiatry and Behavioral Sciences, Northwestern University, , Chicago, IL, United States, United States; 4 Medical Social Sciences, Feinberg School of Medicine, Northwestern University, Chicago, IL, United States; 5 Steve Hicks School of Social Work, University of Texas at Austin, Austin, TX, United States

**Keywords:** assault weapons, FAWB, federal assault weapons ban, firearms, guns, large-capacity magazine, LCM, gun policy, public mass shootings, weapon, weapons, shooting, shootings, prevention, efficacy, surveillance, public health, linear regression, homicide, gun, gun control, gun injury, gun injuries, health policy, information seeking behavior, health informatics

## Abstract

**Background:**

Assault weapon and large-capacity magazine bans are potential tools for policy makers to prevent public mass shootings. However, the efficacy of these bans is a continual source of debate. In an earlier study, we estimated the impact of the Federal Assault Weapons Ban (FAWB) on the number of public mass shooting events in the United States. This study provides an updated assessment with 3 additional years of firearm surveillance data to characterize the longer-term effects.

**Objective:**

This study aims to estimate the impact of the FAWB on trends in public mass shootings from 1966 to 2022.

**Methods:**

We used linear regression to estimate the impact of the FAWB on the 4-year simple moving average of annual public mass shootings, defined by events with 4 or more deaths in 24 hours, not including the perpetrator. The study period spans 1966 to 2022. The model includes indicator variables for both the FAWB period (1995‐2004) and the period after its removal (2005‐2022). These indicators were interacted with a linear time trend. Estimates were controlled for the national homicide rate. After estimation, the model provided counterfactual estimates of public mass shootings if the FAWB was never imposed and if the FAWB remained in place.

**Results:**

The overall upward trajectory in the number of public mass shootings substantially fell while the FAWB was in place. These trends are specific to events in which the perpetrator used an assault weapon or large-capacity magazine. Point estimates suggest the FAWB prevented up to 5 public mass shootings while the ban was active. A continuation of the FAWB and large-capacity magazine ban would have prevented up to 38 public mass shootings, but the CIs become wider as time moves further away from the period of the FAWB.

**Conclusions:**

The FAWB, which included a ban on large-capacity magazines, was associated with fewer public mass shooting events, fatalities, and nonfatal gun injuries. Gun control legislation is an important public health tool in the prevention of public mass shootings.

## Introduction

Public mass shootings constitute a fraction, less than 1%, of the approximately 20,000 annual firearm homicides in the United States [1-5]. However, their notoriety commands national attention, propelling debates on gun policy and fueling the ongoing quest among policy makers to stop these events [6-14]. The US Congress passed the Federal Assault Weapons Ban (FAWB), also known as the Public Safety and Recreational Firearms Use Protection Act, on August 25, 1994, and President Bill Clinton signed the act into law on September 13, 1994 [15]. The ban was set to expire after 10 years in 2004, and Congress did not renew it. The ban prohibited the sale and manufacture of certain semiautomatic weapons and magazines that could hold more than 10 rounds [16]. It also established rules for the secure storage and transfer of firearms and devices regulated by the law that were owned before the legislation.

The definition of an assault weapon can be a source of confusion. Semiautomatic weapons and assault weapons (second grip plus other features) are often mistakenly conflated [17-19]. Semiautomatic weapons will automatically load another cartridge into a chamber but require a handler to manually release and press the trigger to fire each round. Semiautomatic weapons are common in the United States and include the majority of pistols. Automatic weapons further allow a handler to hold the trigger for continuous fire [20]. The FAWB explicitly noted some of the most commonly purchased assault weapons [16]. The ban covered firearms having a detachable magazine and at least two of the following: a telescoping stock, a pistol grip that protrudes conspicuously, a bayonet mount, a flash suppressor, or a grenade launcher. Semiautomatic pistols and shotguns were similarly banned contingent on the presence of other specific attachments.

The FAWB also prohibited the manufacture and sale of large-capacity magazines (LCMs) defined as holding more than 10 bullets [21]. The LCM ban may have been more impactful than the assault weapons ban, as several studies have shown a negative association between LCM bans and casualty counts at the state level [21-26]. These and other studies have also examined the broader effect of the FAWB on various outcome measures [27-29]. For example, Gius [30] found that the FAWB was associated with fewer mass shooting deaths in a model combining state and federal bans, but Koper et al [28] did not find an association between the FAWB and deaths in a broader inclusion of all gun homicides.

This study focuses on the impact of the FAWB on public mass shootings. The approach differs from previous research in three aspects: (1) a focus on public mass shooting events as the primary outcome variable, (2) counterfactual estimates of the number of events that would have occurred had the FAWB never been implemented, and (3) analogous estimates if the FAWB were continued. Because assailants often aim to maximize casualties, the restrictions imposed by the FAWB may have had a greater impact on public mass shootings than on other types of mass shootings (eg, family annihilation) [31,32]. Assault weapons and LCMs facilitate the rapid discharge of rounds, increasing the potential for higher casualty counts [21].

Most FAWB studies focus on the reduction in fatalities or injuries as outcome variables. Koper and Roth [27] and Post et al [33] are exceptions, which focused on the number of public mass shootings. However, Koper and Roth [27] tempered their FAWB research findings “because the ban’s long-term effects could differ from the short-term impacts revealed by this study.” To that end, this study extends our prior research to examine the association between the FAWB and public mass shooting events.

Our previous study followed a similar methodology, and the results indicated an increase in public mass shooting events, fatalities, and injuries following the expiration of the FAWB [33]. This study provides an update with an additional 3 years of data and trend analysis for fatalities and injuries, which were not included in the original study. Lastly, this study also includes results on public mass shooting events separated by those in which a weapon potentially classifiable as an assault weapon was used.

## Methods

### Overview

To define a public mass shooting, we adopted the Federal Bureau of Investigation’s definition of a massacre, in which 4 or more people (apart from an assailant) are killed within a single event [34]. We added the requirement for a shooting to have occurred in a public setting and committed within a 24-hour time frame, as in Fox et al [35-37]. This restriction distinguishes public mass shootings from other types of spree killings, which can occur over longer time and location horizons. Data were sourced from the Violence Project, which maintains a database on mass shooting events in the United States from 1966 onward. The Violence Project is led by Peterson and Densely [38], who make data available through Hamline University.

We used linear regression to estimate the impact of the FAWB on the 5-year simple moving average (SMA) [39,40]. The SMA model estimates the mean value for public mass shooting events for each year:



(1)
$$E_{t}=\beta_{0}+\beta_{1}fawb+\beta_{1}fawb\cdot t+\beta_{2}postfawb\;\;+\beta_{3}\;postfawb\cdot t+\beta_{4}t+\beta_{5}hom_{t}+e_{t}$$

The dependent variable, $E_{t}$, is the 5-year SMA of public mass shootings in year *t*. The indicators fawb and postfawb are set to 1 for the years 1995‐2004 and 2005‐2022. Because the FAWB was enacted in late 1994, we coded 1995‐2004 as years under the ban. Lastly, let $hom_{t}$ denote the homicide rate in year *t*. Because year *t* was almost perfectly correlated with population, the model dropped population as a control to avoid high collinearity. Statistical inference was based on an α level of .05 with heteroskedasticity robust SEs.

We conducted two counterfactual exercises. The first estimated the number of public mass shootings that would have occurred from 1995 to 2004 if the FAWB had not been adopted. The second projected forward the number of events that would have occurred had the FAWB remained in place from 2005 to 2022. The difference between the predicted values from these exercises and the actual number of events provided estimates of the number of events prevented by the FAWB and the number of events created by its removal, respectively.

### Ethical Considerations

This study does not constitute research with human subjects because all data were publicly available. Institutional review board review was therefore unsolicited. This study followed the ethical guidelines of the Committee on Publication Ethics and the World Medical Association Helsinki Declaration.

## Results

The data contained 184 public mass shooting events from 1966 to 2022. The years before the FAWB (1966‐1994) contained 55 events. The period of the ban is defined as 1995‐2004 because the legislation was passed at the end of 1994 and expired at the end of 2004. This period contained 34 events, and the period after the ban (2005‐2022) contained 95 events.

**Figure 1.**
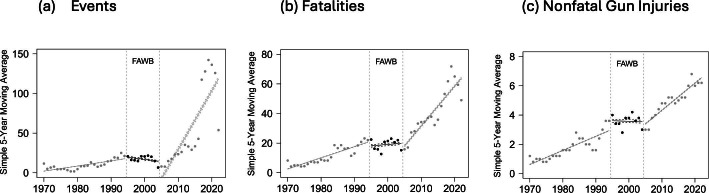
Trends in the 5-year moving average of events, fatalities, and nonfatal gun injuries from public mass shootings in the United States from 1966 to 2022. FAWB: Federal Assault Weapons Ban.

Figure 1A plots the 5-year SMA of events over the sample period. The first data point therefore begins in 1970. The figure shows an increase in events over time. The maximum 5-year SMA of 6.8 occurred in 2019. However, trend lines vary significantly for the periods before, during, and after the FAWB. In particular, the trend was negative for the FAWB period but positive before and after the ban. Figure 1B presents analogous trends for the 5-year SMA of fatalities in public mass shootings, while Figure 1C presents trends for nonfatal gun injuries. The trend line for fatalities was slightly positive during the FAWB, but the magnitude of the slope was much lower than for either period around the ban. The trend line for injuries sloped down during the FAWB, while it sloped up in either period around the ban.

The trend lines in Figure 1 are based solely on year as a covariate. With a focus on events, Table 1 presents the results from the full regression model 1. The ordinary least squares regression fit line returned a slope coefficient of 0.10 for the years 1966‐1994. While the FAWB was in place from 1995 to 2004, the slope was −0.06. The slope became positive again after the removal of the ban. In fact, at 0.20, the slope was nearly twice the magnitude of the period before the ban. The adjusted *R*^2^ value of 0.95 is a common feature of time series analysis, in which *R*^2^ is typically much higher than in a cross-sectional analysis [41].

**Table 1.** Ordinary least squares regression results for model 1.

	$E_{t}$ (SE)	*P* values
*fawb*	327.00 (109.94)	<.001
*postfawb*	−201.92 (42.60)	<.001
fawb⋅t	−0.16 (0.06)	<.001
postfawb⋅t	0.10 (0.02)	<.001
*year*	0.10 (0.01)	<.001
*hom*	−0.23 (0.08)	.008
*constant*	−188.50 (28.16)	<.001
n	53	—^a^
Adjusted *R*^2^	0.95	—
*F* statistic (*df*)	137.32 (6,46)	<.001

a Not applicable.

**Figure 2.**
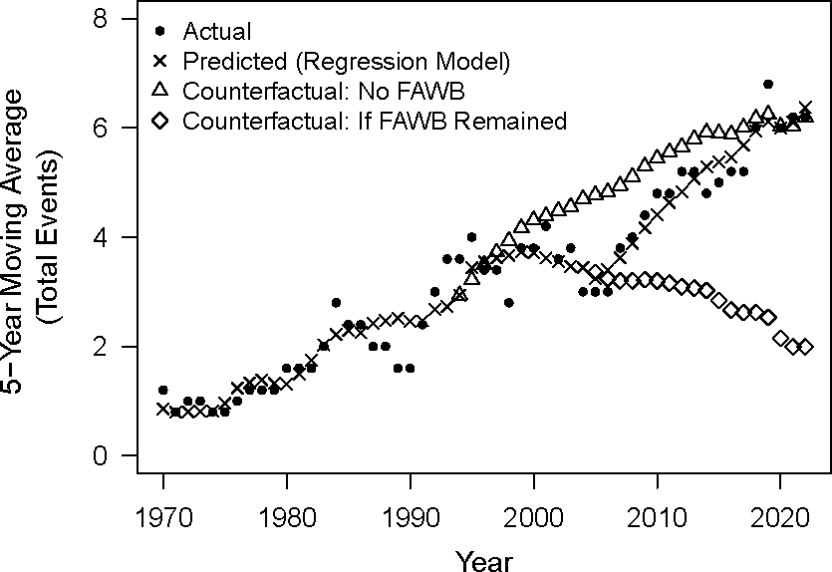
Counterfactual estimates for the number of public mass shootings in the United States from 1966 to 2022 in the absence or continuation of the FAWB. FAWB: Federal Assault Weapons Ban.

Figure 2 presents the counterfactual exercises from the regression model. The first counterfactual trend shows the estimated 5-year SMA of events if the FAWB had never been imposed. Estimates are denoted by triangles, and they are much higher than the actual moving average of events from 2000 until roughly 2021. The exercise indicates a substantial increase in events if the FAWB had not been imposed. The sum of the annual differences between the counterfactual and actual SMAs from 1995 to 2004 suggests the FAWB prevented 5 public mass shootings.

The second counterfactual exercise shows the estimated 5-year SMA of events if the FAWB had continued until 2022. Estimates are denoted by diamonds. The results starkly diverge from the actual moving average of events. The counterfactual estimates began an immediate downward trend after the FAWB, while the actual moving average quickly trended upward. The sum of the annual differences between the counterfactual and actual moving averages from 2005 to 2022 suggests that a continuation of the FAWB may have prevented up to 38 events over the period (see Multimedia Appendix 1 for Figure 2 with 95% CIs).

**Figure 3.**
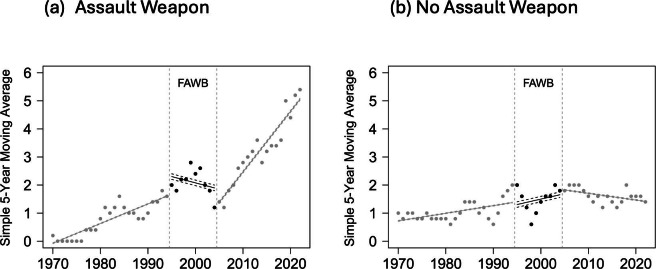
Trends in the 5-year simple moving average of public mass shootings in the United States from 1966 to 2022 that did and did not involve an assault weapon. FAWB: Federal Assault Weapons Ban.

Data from the Violence Project also contain detailed information on the firearms used in events. Figure 3 contains trend lines, similar to Figure 1, but for events in which either an assault weapon was used or not. To derive these categories, we first set a filter to exclude weapons collected by police but recorded as not used in the shooting. We coded “yes” for an assault weapon whenever at least one weapon in the event was designated as either an assault weapon or had an LCM. However, for every revolver, we reclassified any missing value for large capacity to “no,” as these firearms cannot be modified to have a large capacity. We also consulted with firearm experts to classify the remaining missing values in the database. Of the 192 public mass shooting cases, 116 involved at least one assault weapon, and 76 did not involve any. We noted that these categories should be viewed as proxies to coverage under the FAWB given the complexity of the legislation (see the Introduction section for details on the types of weapons covered by the ban).

The results are consistent with the hypothesis that the FAWB reduced public mass shooting events. In Figure 3A, the SMA of events in which the perpetrator used at least one assault weapon trended upward outside of the FAWB and downward during the FAWB. In comparison to Figure 3B, trends in the SMA for events in which no assault weapon was used were relatively flat, and the magnitudes of the SMAs were much smaller.

## Discussion

### Principal Findings

The results suggest the FAWB had a sizeable impact on the number of public mass shootings in the United States. From 1966 to 2022, public mass shootings trended upward. However, the trend was interrupted while the FAWB was in place. Furthermore, counterfactual exercises suggest events would have been higher if the FAWB had not been imposed, events would not have risen so rapidly after 2004 if the FAWB had remained in place, and mass shooters without access to an assault weapon and LCMs did not substitute other weapons to commit mass shootings. Our first counterfactual estimate is consistent with but smaller than Nagin et al’s [12] and Koper et al’s [20,24,27,28] findings: 5 versus 9 fewer events during the 10-year FAWB. DiMaggio et al [42] also reported a decrease in events during the ban using a shorter sample period with fewer events. Gius [30] found that the FAWB was associated with fewer mass shooting deaths in a model combining state and federal bans. While Koper and Roth [27] and Koper et al [28] did not find an effect, the absence may be explained by the inclusion of all gun homicides, whose substantially higher numbers could wash out any effect on the subset of public mass shootings. The triangulation of these results is meaningful because each study relied on different data sources and statistical approaches.

Compared to our first study, the second counterfactual estimate of how many mass shootings could have been prevented if the FAWB remained in place is greater (38 vs 30 fewer events). However, this increase is driven exclusively by a longer sample period, as the additional 3 years of data arrived when the rate of events was at a record high. Finally, the results in Figure 3 show a substantial increase in the rate of events in which an assault weapon was used after the FAWB was lifted.

Two points from our initial study deserve repetition. First, the increase in public mass shooting events cannot be attributed to population growth, as the rate of events has outpaced population growth. The US population grew by approximately 70% from 1966 to 2022, while the 5-year moving average of events more than quintupled [43]. The regression results were also controlled for year, which is almost perfectly collinear with the population over the sample period. Second, the negative sign on the homicide rate covariate implies the rise in public mass shootings is not simply a function of the overall homicide rate.

### Mechanisms of Action

The FAWB may have worked through its two primary mechanisms: a ban on assault weapons and a ban on LCMs. For example, Webster et al [26] and Klarevas et al [21] found that state LCM bans were associated with fewer public mass shootings and deaths per event. Because our study focused on the FAWB, we cannot differentiate between the two mechanisms of action. We note, however, that both mechanisms can affect the ability of an assailant to maximize death counts in public mass shootings. Both may therefore be important deterrents.

A decrease in fatal and nonfatal gun injuries during the FAWB is intuitive because assault weapons combined with LCMs enable mass shooters to rapidly discharge dozens of rounds within seconds. The use of an assault rifle with an LCM doubled fatalities and increased nonfatal gun injuries by 81% compared to public mass shootings without these weapons [44]. Moreover, Koper [24] and other investigators found that fatal mass shootings involving LCMs had 60%‐67% higher fatality counts than those without [21,45]. What is less intuitive is why the FAWB resulted in fewer mass shooting events. Why would the ban dissuade mass shooters from committing mass shootings in the first place? One possible explanation is the desire among mass shooters to maximize death and injury, which is hindered by assault weapon and LCM bans. Another explanation is that mass shooters may prefer the active intimate role in homicide offered by a firearm over a more passive approach, such as a bomb or arson. Furthermore, Fox and Levin [31] identified a large proportion of pseudo-commando–themed mass shootings where mass shooters dressed in battle fatigues, which included assault weapons. Some mass shooters may be driven more by the desire to project an image of power and control than by the outcome of their actions. These individuals often seek to demonstrate masculinity through symbols of military prowess, adopting a “pseudo-commando” persona to emulate military operations. If the means are as important as the end, the accessories of power and control, including assault weapons and LCMs, become necessary for these shooters. Finally, perpetrators may want to emulate prior events in terms of weapons and locations, and an increasing number of high-profile events involve the use of an assault weapon.

### FAWB Limitations

The FAWB carried several limitations. First, the ban contained a grandfather clause in which any previous owner of banned weapons was allowed to retain them [46]. Second, many weapons remained in the community because the ban was unaccompanied by a buyback program [28]. Third, gun legislation allows buyers who acquire weapons from gun shows or directly from another owner are not required to pass background checks [47,48]. While guns are registered at the point of sale from an arms dealer, most states do not regulate the transfer of arms from one owner to the next, nor do they require a background check [49-52]. The effects of the FAWB may have been stronger without these limitations. For example, Australia, England, Canada, and New Zealand implemented gun buyback programs that substantially reduced gun deaths [53-57].

### Potential Confounders

This study extends our prior research by incorporating an additional 3 years of data, spanning from 2020 to 2022, a period that coincides with the onset and progression of the COVID-19 pandemic—the largest global health crisis in recent history [58,59]. The COVID-19 pandemic likely curtailed public mass shootings because its onset corresponds with the conspicuous absence of public mass shootings. Every database tracking public mass shootings shows an increase of public mass shootings over time with a notable dip at the start of the COVID-19 pandemic [60-63]. The last public mass shooting occurred on March 15, 2020, in Springfield, Missouri, 1‐3 weeks before state government agencies imposed mandatory stay-at-home orders [64]. Subsequently, the United States experienced a hiatus of 10 months, or 300 days, devoid of public mass shootings—a remarkable departure from the preceding two decades [60,61]. Several factors likely explain the interruption. For example, stay-at-home orders, social distancing, quarantines, and bans on large gatherings may have reduced opportunities to successfully carry out a mass shooting. The rate of events rebounded in 2022 to a level consistent with the rate at the onset of the pandemic. Lastly, other confounders may also be present, such as growth in the manufacture of guns or the evolution of media coverage around mass shootings.

### Limitations

This study found a statistically significant difference in the incidence of public mass shootings during the FAWB. However, because the assault weapon and LCM bans in the FAWB occurred simultaneously, we cannot separately analyze the impact of one component of the legislation from the other.

Several limitations also stem from the long time horizon of the study, in particular from the reduced ability to track the implications of the FAWB as time moved further away from the sunset of the legislation. For example, due to data availability, estimates did not control the manufacture and sale of firearms in the United States, but an escalation of firearm sales coincided with the end of the FAWB [65]. Part of this increase is explained by the removal of the FAWB, but part of the increase is explained by external factors. The estimates do not control for changes in media saturation over time. “New media” had displaced mass communication while the FAWB was in place. The adoption of new media continued to expand substantially after the legislation expired [66,67]. This shift may be important because the internet is a likely conduit for mass shooters to become famous through additional mass communication channels, research and emulate prior events, connect to other extremist individuals, and learn how to plan attacks [68-71]. These factors may have contributed to the growth in mass shootings over time.

### Conclusion

Public mass shootings are a unique type of firearm homicide [32]. These events may respond to different factors and policies than other types of firearm homicide [32]. Building on research conducted shortly after the FAWB ended, our study corroborates the impact that the FAWB had on mitigating the frequency of public mass shootings during its enforcement period. The analysis indicates that a continuation of the FAWB would have reduced the rate of public mass shootings since 2005. Although a federal ban will not eliminate all public mass shootings, the results of this study indicate that a ban can meaningfully alter the trajectory of gun violence over time.

## Supplementary material

10.2196/62952 Multimedia Appendix 1 Counterfactual estimates with 95% CIs for the absence and continuation of the Federal Assault Weapons Ban.

## Abbreviations

FAWB: Federal Assault Weapons Ban
LCM: large-capacity magazine
SMA: simple moving average
